# Listening to the Human Voice Alters Sensorimotor Brain Rhythms

**DOI:** 10.1371/journal.pone.0080659

**Published:** 2013-11-12

**Authors:** Yohana Lévêque, Daniele Schön

**Affiliations:** 1 Laboratoire Parole et Langage, Centre National de la Recherche Scientifique (CNRS) and Aix-Marseille Université, Aix-en-Provence, France; 2 Institut de Neurosciences Cognitives de la Méditerranée, Centre National de la Recherche Scientifique (CNRS) and Aix-Marseille Université, Marseille, France; Harvard Medical School/Massachusetts General Hospital, United States of America

## Abstract

While neuronal desynchronization in the mu (^≈^10Hz) and beta (^≈^20Hz) frequency bands has long been known to be an EEG index of sensorimotor activity, this method has rarely been employed to study auditory perception. In the present study, we measured mu and beta event-related desynchronisation (ERD) while participants were asked to listen to vocal and triangle-wave melodies and to sing them back. Results showed that mu and beta ERD began earlier and were stronger when listening to vocal compared to non-vocal melodies. Interestingly, this humanness effect was stronger for less accurate singers. These results show that voice perception favors an early involvement of motor representations.

## Introduction

Imitation is one of the key mechanisms in the acquisition of infants’ motor and vocal skills. In particular, vocal imitation shapes the auditory-motor associations that will enable the infant to control their vocal production in order to sing and speak. In humans, exploration into the auditory-vocal loop is still in its early stages. Nevertheless, neuroimaging has revealed activations in the human motor system which occur during the perception of actions and, in particular, during speech perception. For instance, blood-oxygen-level-dependent responses in Broca’s area and the premotor cortex have been found during passive listening of speech [1-3 and others]. At the peripheral level, measurement of motor-evoked potentials following Transcranial Magnetic Stimulation revealed an enhanced excitability of the listener’s tongue muscles when listening to syllables involving tongue movement [4, 5]. Although the functional role of these activations is still debated (e.g. [6]), this motor activity seems to affect the performance in tasks such as phoneme discrimination or speech in noise perception [7]. 

Few studies have investigated the production-perception link using vocal but non-linguistic stimuli. Chang et al. [8] suggested that non-linguistic laryngeal sounds like cough or laughter activate the same motor regions as speech. Studies of Warren et al. [9] and Meyer et al. [10] showed that listening to emotional vocalizations induced premotor activations in the larynx/mouth region of the bilateral premotor cortex. Similarly, Schön et al. [11] reported an activation of the premotor and (more marginally) orofacial sensorimotor cortex during perception of sung syllables. Finally, Brown and Martinez [12] showed that vocal-motor planning areas could be activated by a task of melody discrimination, while no such activity was reported during passive listening of melodies.

In the visual modality, a set of studies from the last decade suggests that activity in motor or premotor regions during perception is specific to or stronger for biological or human-produced stimuli. They showed that, compared to human movements, motor areas have a weaker activity when the perceived movements are impossible for a human being [13], performed by a robot [14, 15], scrambled [16], or specific to animals [17]. In the auditory modality, Galati et al. [18] reported that listening to human action sounds after congruent semantic priming elicited activations in the “auditory mirror system”, while it was not the case for environmental sounds. This “humanness bias” could be explained by the fact that non-human stimuli are not bound to body representations as strongly as human-produced stimuli, and so do not activate strong sensorimotor connections. In the case of voice, while selective brain areas [19] in the temporal lobes and characteristic evoked potentials [20-23] have been reported, the role of sensorimotor representations in voice perception has not been directly addressed. 

The aim of the present work was to compare the involvement of the sensori-motor system when listening to a singing voice or to similar non-vocal melodies. At this aim we focused on EEG brain rhythms (oscillations) known to be related to bodily movements: beta (typically around 20Hz) and mu (around 10Hz). Previous studies have demonstrated an Event-Related Desynchronization (ERD) of neuronal groups in these frequency bands during realization of limb or mouth movements (see [24] for a review). Additionally, it has been shown that an ERD in the beta frequency band precedes the production of a voluntary movement by 1 to 2s, indicating motor preparation [25- 27], while there is an event-related synchronization (ERS) once the movement is completed. Generators of these rhythms have been localized principally in the precentral cortex for beta and the postcentral cortex for mu [28 or 29] as confirmed by a study coupling EEG and fMRI [30]. Interestingly, Gunji et al. [31] showed that vocal production also induces a similar ERD, even without articulatory mouth movement (humming). 

In addition to studies investigating movement production, beta and mu ERDs have been used to detect and characterize sensorimotor activity during action observation for a long time [32- 35]. By contrast, desynchronization of beta and mu rhythms during action sound perception has been studied only recently, with a few works on music perception [36, 37], action sounds [27, 38] and speech [39-41]. To our knowledge, no one has yet investigated electrophysiological correlates of a potential motor system involvement in the perception of a singing voice. 

In the current study we tested the hypothesis that perceiving a singing voice would induce a stronger sensorimotor activity, i.e. a beta and mu ERD, than perceiving a non-vocal melody. We used an imitation task, which means that participants were listening to the melodic models with the intention to sing them back afterwards. Based on the existing literature, we expected to observe (a) an ERD in the mu and beta frequency bands during vocal production, in line with Gunji et al. [31], and (b) a larger beta and mu ERD during the perception of singing voice compared to the perception of the non-vocal sounds. These results would imply that, in a repetition task, hearing a human voice activates the motor representations required to produce the sound more strongly than a non-vocal model. This is in line with what the motor theory of speech perception suggested: by positing a strong link between speech perception and production [42], it predicts a facilitated engagement of the motor representations for articulatory sounds. 

## Materials and Methods

### Participants

We tested 20 right-handed adults with no history of neurological, voice or hearing disorders (28.8 ± 6.6 yrs; 10 females). Participants had an average of 5 years of musical training (SD=6). Three participants also sung in a choir for two years during childhood. The experiment was conducted in accordance with the Declaration of Helsinki and after ethics committee approval (CNRS - Institut de Neurosciences Cognitives de la Méditerrannée). All subjects gave written informed consent to participate to the experiment and received a chocolate box as a present. EEG analyses were performed on a sample of 19 participants, after one participant was discarded due to acquisition problems. Acoustic analyses were performed on a sample of 18 participants due to a technical problem with one subject.

### Experimental Setup and Stimuli

One hundred and twenty isochronous melodies of five 750-ms notes were composed by using a pseudo-random concatenation of pitches, taking into account the female and male pitch ranges (tessitura), avoiding intervals greater than 10 semi-tones and favoring small over big intervals as is typically the case in song repertoire [43]. Repetition of a given tone was allowed. Melodies for a female tessitura were comprised between G3 and G4, and transposed to the range C3-C4 for a male tessitura. The 120 melodies were recorded in an anechoic room, by a man and a woman, singing on the vowel /o/. Recordings were done using a Sennheiser PC131 microphone amplified by a Zoom H16 station and digitized using SoundForge software at 44.1 kHz. Singers sang along with a metronome presented via headphones at 80 beats per minute and were asked to sing one note per beat. One hundred and twenty identical melodies were also generated using a computer-generated sound built by superposition of the fundamental and the first three harmonics with decreasing intensity, using a triangular wave at sampling rate of 44.1 kHz. An envelope of 5 ms-rise and fall time was applied to each sound and stimuli were normalized in intensity using Adobe Audition. Note that a triangle-wave timbre was chosen rather than an instrument timbre, in order to prevent a motor resonance linked to possible previous practice of musical instrument.

**Figure 1:**
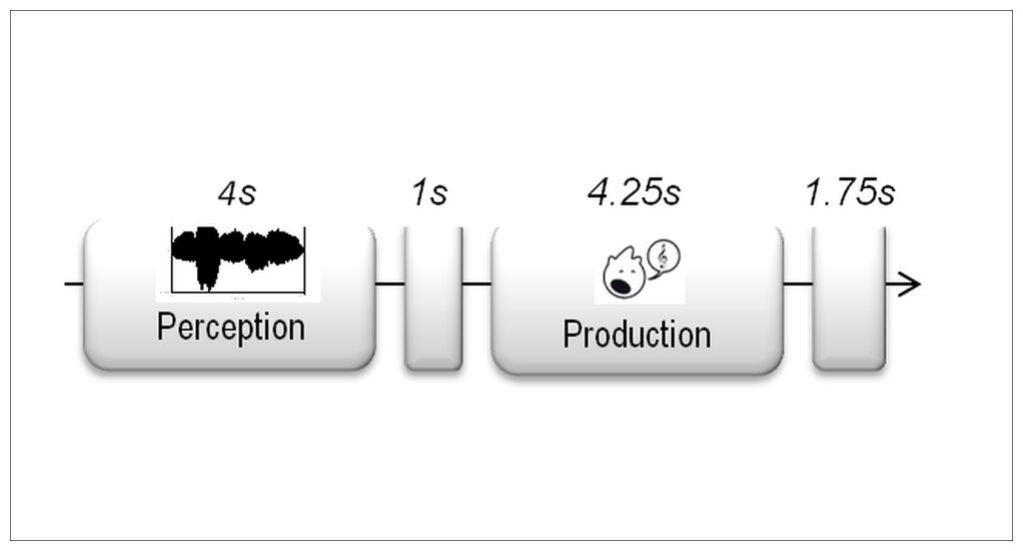
Summary of task protocol. Participants listened and repeated short melodies. They were instructed to fix the center of the screen, and to breathe in and sing when the Go signal appeared.

Sung notes had an average jitter (varying pitch) of 0.29%, a shimmer (varying amplitude) of 1.35%, a fundamental frequency (*f0*) standard-deviation of eight point five cents and a median note duration of 0.738 ms while computer-generated notes had an average jitter and shimmer inferior to 0.05%, an f0 standard-deviation inferior to one cent and a note duration of 0.750ms. The median pitch deviation between the vocal and computer-generated models was of thirteen point five cents of a semi-tone, which reflects the good pitch accuracy of the vocal models. The task was programmed and presented using E-Prime 2.0 (Psychology Software Tools, Inc.). Half of the melodies were presented with the vocal timbre and half with the non-vocal timbre. Assignment of a melody to a given condition (timbre) was counterbalanced across subjects and the order of presentation of the 120 melodies was randomized. Participants, sitting on a chair in an electrically and acoustically shielded room, received the instruction to listen to each melody (earphones Shure SE115) and sing it back only after a visual GO signal was presented on a computer screen. The timing of the procedure is illustrated in Figure 1. Participants were asked to keep their body relaxed, to fixate the center of the screen, and to take their phonatory inspiration after the visual signal to avoid anticipations and oro-facial movements during the perception phase. The experiment began with 5 practice trials. A pause took place every 30 trials. Participants’ vocal production was recorded with a microphone PHM903 AV –Jefe and Audacity software (http://audacity.sourceforge.net/).

### EEG recordings

Continuous EEG was recorded through the ActiveTwo BioSemi electrode system (BioSemi, Amsterdam, the Netherlands) from 64 electrodes located at standard left and right hemisphere positions over frontal, centro-parietal, parietal, parieto-occipital, and temporal areas (International 10/20 system sites) and sampled at 512Hz. The data were then re-referenced ofﬂine to the algebraic average of the left and right mastoids. One further channel on the superior orbicularis muscle was used to measure the mouth’s electromyographic (EMG) activity. A webcam allowed the experimenter to monitor participant’s movements. 

### Data Analysis

#### Acoustical analysis

The recordings of the participants’ vocal productions were manually segmented in Praat speech processing software. Boundaries were placed between the sung notes, including the vocally unstable phases in the notes. The median fundamental frequency (*f0*) of each segment (note) was automatically extracted using a Praat script (5 *f0* values per melody). Four interval sizes per melody were then computed in cents of a semi-tone, for a total of 480 intervals per participant. We then compared these produced interval values to the target interval values, and obtained an average absolute *f0* deviation or error value for vocal and non-vocal conditions. The lower the deviation from the target, the better the vocal accuracy. 

#### EEG analysis

 EEG data were analyzed offline using EEGLAB toolbox [44]. Signals were filtered with a highpass short IIR filter (1Hz). Artifact rejection of continuous data was carried out using independent component analysis (ICA) which allowed separation of eye blinks from the brain signal [45]. After rejection of components representing vertical and/or horizontal eye movements, the signal was reconstructed from the remaining components. Continuous EEG data was then segmented into 6-s epochs for perception periods (1 s before and 5 s after the stimuli onsets), and into 7-s epochs for production periods (1 s before and 6 s after the GO signal). Artifacts due to muscular activity were removed from the segmented signal using the Automatic Artifact Removal toolbox version 1.3 [46] based on a BSS algorithm (Blind Source Separation). Epochs containing extreme values (above 100 uV), improbable data (above 5 SD), and abnormally distributed data (kurtosis above 5 SD) were also rejected. On average, analyses were thus performed on 91/120 epochs for production (SD=17), 51/60 epochs for vocal melody perception (SD=4.8) and 53/60 epochs for non-vocal melody perception (SD=3.7). 

Analyses were performed on 6 regions: fronto-central right (F2, F4, FC2); fronto-central left (F1, F3, FC1); centro-parietal right (C2, C4, CP2); centro-parietal left ( C1, C3, CP1); parieto-occipital right (PO4, PO8, O2); parieto-occipital left (PO3, PO7, O1). Centro-parietal and fronto-central regions were hypothesized to reflect motor system activity. The two parieto-occipital regions were added as control regions. 

Using the Welch’s periodogram, the average power spectral density was first computed as followed. Each epoch comprised several 512-point Hann windows (i.e. 1 sec). Output consisted of 256 amplitude estimates with a frequency bin width of 0.5 Hz. The transformation was calculated separately for each participant, channel and condition and was averaged within two separate frequency bands: mu (7.5-12Hz) and beta (14-21 Hz). On the basis of an exploratory study we knew that ERD was limited to the mu and low beta (up to 21 Hz). Moreover, low beta is less susceptible than high beta to residual muscular artifacts that may be present during production. The signal was analyzed from 0 to 3500 ms after stimulus onset for the perception period and from 0 to 5000 ms after the signal to sing for the production period. The mean power spectral density in the pre-stimulus period (from -500 to 0 ms) was considered as the baseline level and subtracted from each window analysis for each frequency band. 

First, mu and beta power was analyzed using a three-way repeated measures analysis of variance (ANOVA) with the following factors: Condition (production/perception), Laterality (left/right), Anteroposterior gradient (fronto-central, centro-parietal, parieto-occipital). Post-hoc comparisons were estimated using the Tukey’s HSD test.

Second, we compared rhythmic activity during perception of non-vocal and vocal melodies, running an ANOVA with the following factors: Humanness (vocal versus non-vocal melodies), Laterality (left/right), Anteroposterior gradient (fronto-central, centro-parietal, parieto-occipital). P-values were adjusted using the Greenhouse-Geisser correction when appropriate.

 We then used the sLORETA software [47] to localize the source of the response difference to vocal and non-vocal melody perception in the two frequency bands. The analysis was conducted on the perception epochs with a three-shell spherical head model registered to the Talairach atlas (voxel dimension 5 x 5 mm). Statistical significance was assessed by means of a nonparametric randomization test (p<0.05, FDR corrected for multiple comparisons). 

 Finally, in order to gather more precise information on the influence of the factor Humanness on the temporal evolution of mu and beta rhythm, the time course during both the stimulus perception and response preparation (i.e. silence before the go signal) was cut into 5 time windows (1s each) from stimulus onset to the Go signal (thus including the post stimulus silence). Based on the results of the first analysis, the average spectral power was computed within the fronto-central region (F2, F4, FC2, F1, F3, FC1) in the mu and beta frequency bands for each window. A repeated-measures ANOVA was then performed with factors of Humanness (Vocal versus Non-vocal melody listening) and Time (5 windows), followed by a Fisher’s LSD post-hoc test on the significant interactions.

## Results

### Singing Production

In the mu/alpha frequency band, a lower power was found in the Production phase compared to the Perception phase (F(1,18) = 6.002, p=.025), with no interaction with the Anteroposterior gradient (F(2,36) =0.3, p=.74) nor with the Laterality factor (F(2,36=0.12, p=.73). Note that brain rhythm around 10 Hz is typically called alpha rhythm, but more specifically “mu” rhythm when its localization is centro-parietal or fronto-central, maximal over the sensorimotor cortex at rest [29].

In the beta band, too, a lower power was found in the Production phase compared to the Perception phase, this time interacting with the Anteroposterior gradient (F(2,36) = 4.9 , p=.013) but not with the Laterality factor (F(2,36=0.04, p=.85). More specifically, the difference between production and perception was significant over centro-parietal but not over fronto-central and parieto-occipital regions (fronto-central: p=.25, centro-parietal: p=.003, parieto-occipital : p=.99). 

**Figure 2:**
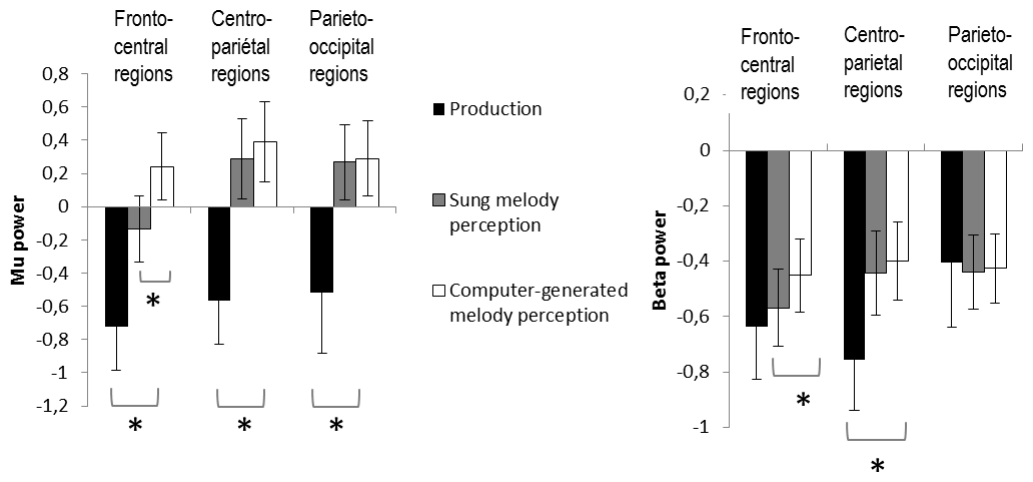
Beta and mu suppression in the experimental conditions. Bars represent the mean power in the beta (14-21Hz) and mu frequency bands (7.5-12Hz) during the three conditions (Production, Vocal melody perception, Non-vocal melody perception), over the power in the baseline, for the fronto-central, centro-parietal and parieto-occipital regions. Data from the left and right hemispheres are averaged. Error bars indicate the standard error of the mean and asterisks correspond to the significant differences (p<.05) between conditions (Bottom: Production versus Perception, Top: Vocal melodies versus Non-vocal melodies), according to Tukey’s HSD post-hoc tests. Note that differences between vocal and non-vocal in the beta band were only significant over left fronto-central electrodes.

This first analysis thus confirmed that vocal production was associated to a clear power decrease in the beta and mu band (Figure 2). As expected, analysis of the EMG signal at the labial electrode also revealed a greater muscular activity during production compared to perception in the frequency band 10-100 Hz (t(18)=3.35, p<.001). 

### Vocal and non-vocal melody perception

In the mu band, the ANOVA comparing Vocal melody perception to Non-vocal melody perception showed a significant effect of Humanness (F(1,18) = 4.44, p=.049), and a significant interaction Humanness*Anteroposterior gradient (F(2,36) = 17.67, p<.001). Post-hoc comparisons revealed a greater ERD during Vocal melody perception compared to Non-vocal melody perception in the fronto-central (p<.001) regions (centro-parietal regions: p=.19, parieto-occipital regions: p=.99). There was no interaction between Humanness and Laterality (F(2,36)=0.32, p=0.58).

 In the beta band, we found a significant three-way interaction Humanness*Laterality*Anteroposterior gradient (F(2,36) = 4.36, p=.035). Post-hoc comparisons revealed that the effect of Humanness was significant in the left fronto-central region (Left fronto-central: p<.001, Right fronto-central: p=.19, Left centro-parietal: p=.95, Right centro-parietal: p=.55, Left parieto-occipital: p=.99, Right parieto-occipital: p=.99). Again there was no effect of laterality.

**Figure 3:**
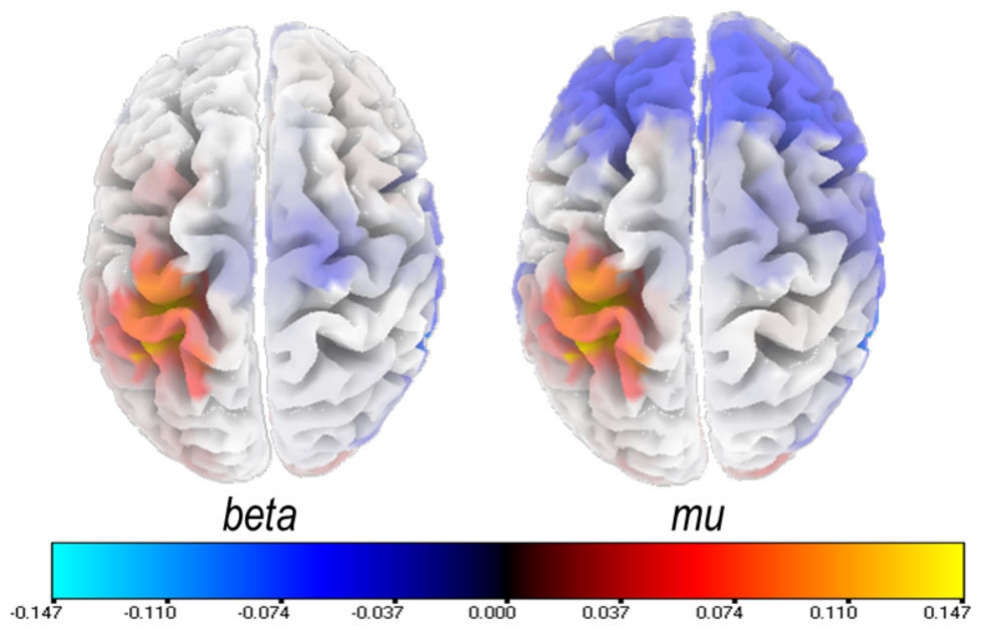
LORETA differences between vocal and non-vocal melodies during the perception epochs in mu and beta frequency bands. Statistical non-parametric current density maps. Color code indicates log of F-ratios. Positive values (red) indicate larger ERD for vocal compared to non vocal. Note that stimuli acoustical features did not explain mu and beta ERD. Indeed, we computed for each vocal stimulus the mu and beta power values, averaged across subjects over the fronto-central region. We then ran separate multiple regressions for mu and beta measures including as predictors: jitter, shimmer, f0 variation and median note duration for each male or female sung melody. Results show that these variables did not explain the variance of mu and beta activity: R^2^ of the multiple regressions did not exceed 0.03, with F(4, 115)<0.9 and p>0.05. None of the 4 factors reached significance individually.

Figure 2 shows average mu and beta power minus the baseline for Production and the two perceptive conditions. The sLORETA source localization indicated that voice perception specifically activated the left superior sensorimotor region (Figure 3) compared to perception of non-vocal melodies.

**Figure 4:**
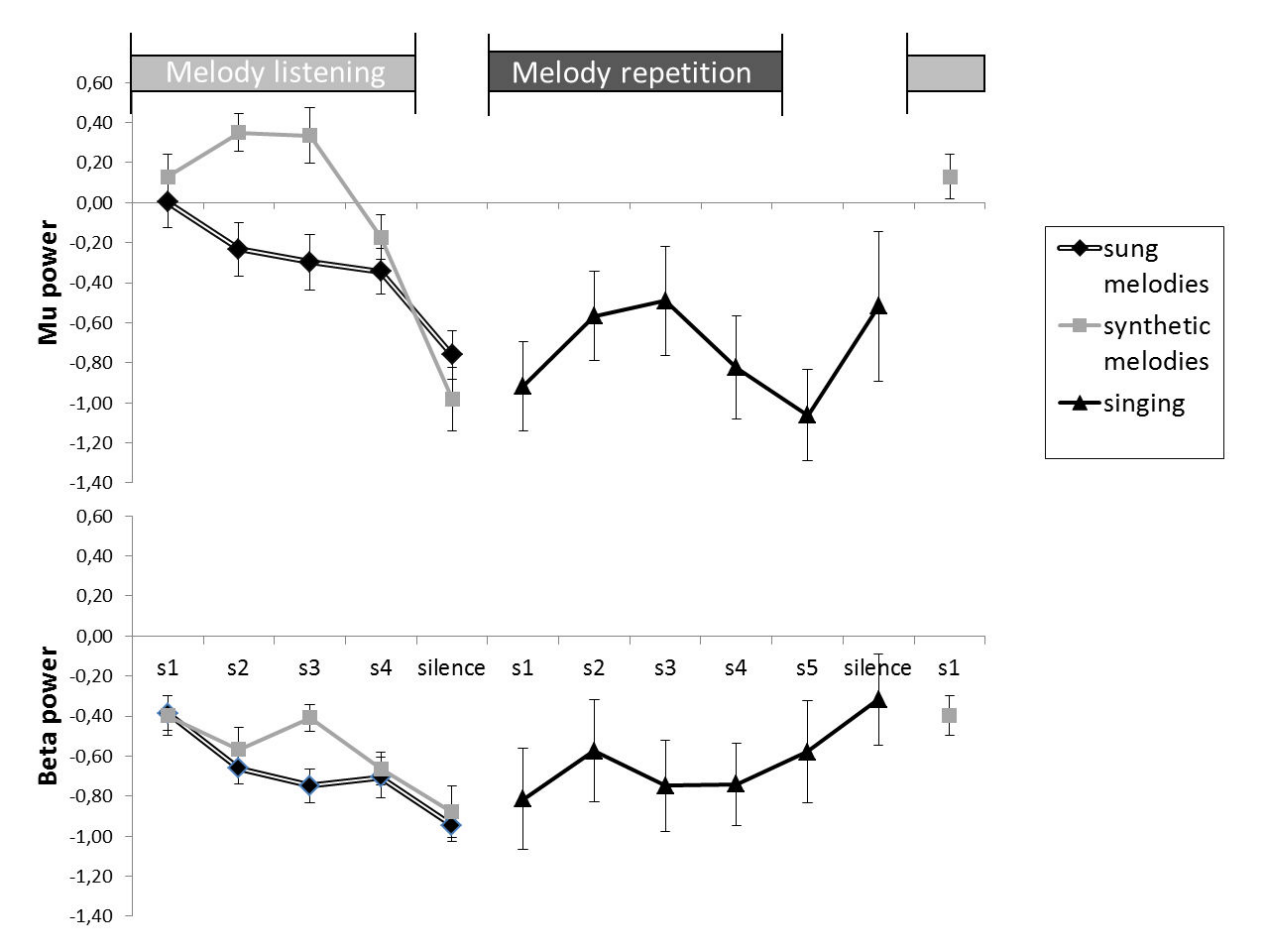
Time course of mu and beta activities over fronto-central electrodes during melody listening and production. Errors bars denote the within-subject confidence intervals.

Figure 4 illustrates the average time-course of mu and beta power along a trial in the fronto-central region, where the humanness effect was significant. In order to test the interactions between humanness and time, a repeated measures ANOVA was performed on fronto-central rhythmic activity (F2, F4, FC2, F1, F3, FC1) during perception, with factors of Humanness (Listening to vocal versus non-vocal melodies) and Time (5 windows of 1 second each). The Humanness*Time interaction was significant for mu rhythm (F(4,72)=4.87, p=.006), with a significant humanness effect in the 1 to 2 s (p<.001) and 2 to 3 s post-onset windows (p<.001) as shown by a post-hoc LSD test. A tendency toward a main effect of humanness was found for beta rhythm (F(1, 18)=3.74, p=.069), as well as a time effect (power decrease across time F(4, 72)=5,12, p=.003). Interestingly, post-hoc comparisons showed that the effect of humanness was significant at time bin 3 only (i.e. between 2 and 3 s, p=.003). 

We found no significant difference in labial EMG activity between perception of vocal and non-vocal melodies (t(18)=0.42; p=.68). 

### Acoustical analysis results

 A Wilcoxon test comparing participant's accuracy in singing back the melodies after vocal versus non-vocal models showed no clear effect of sound humanness (median deviation to the target interval: 52 cents for vocal melodies, 60 cents for non-vocal melodies; W=54, p=.18). 

**Figure 5:**
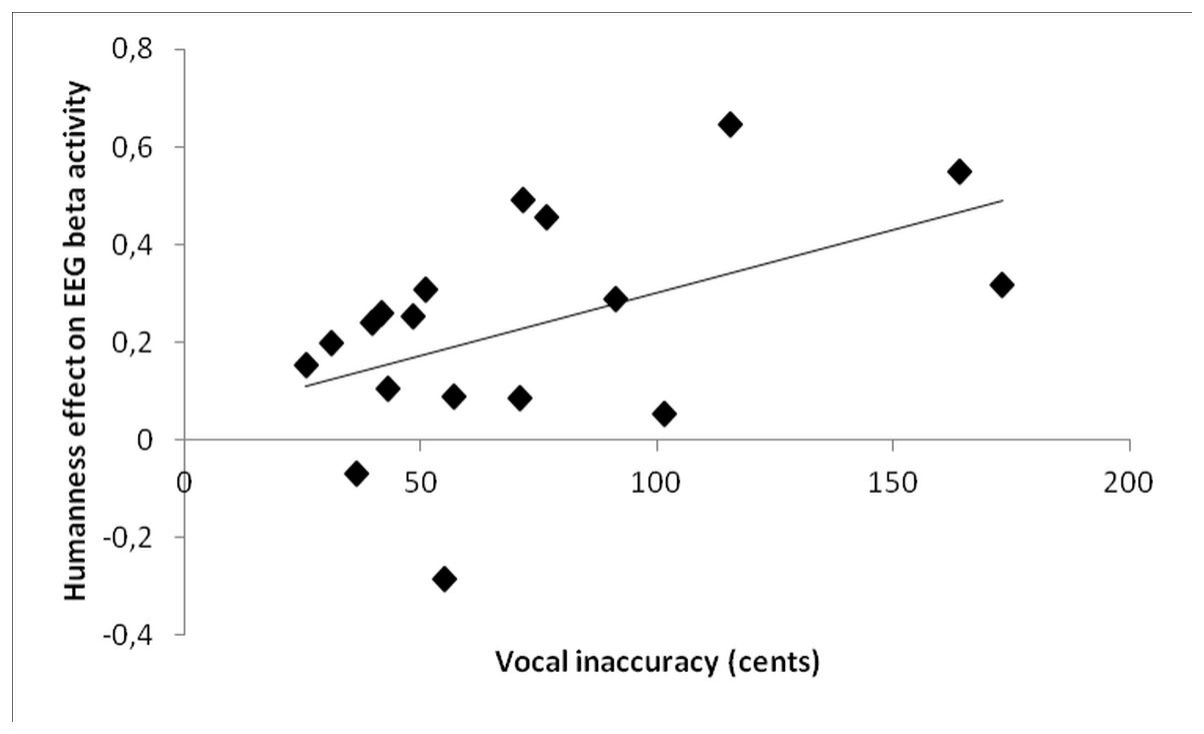
Relationship between vocal inaccuracy and ERD in the beta band for voice listening. Vocal inaccuracy corresponds to the mean deviation from the target pitch intervals in cents of a semi-tone. Humanness effect is computed by subtracting beta power in the non-vocal condition to the beta power in the vocal condition, averaged across 6 fronto-central electrodes, in the window from 250 to 3000 ms after the stimulus onset.

 Examining the relationship between beta and mu power and our acoustic measures, we found a significant correlation between mean vocal accuracy and the voice-specific beta activity found over the fronto-central region (beta power during non-vocal melody perception minus beta power during vocal melody perception): Spearman coefficient r=.49, p=.03 (Figure 5). 

## Discussion

 Assuming that a decrease in power of mu and beta-motor rhythms indicates sensorimotor activity in the brain, these oscillatory activities were studied while participants performed a singing imitation task including vocal and non-vocal melodies. First, consistent with Gunji et al. [31], vocal production involved a significant ERD in mu and beta bands in the fronto-central region. Second, during the perception phase, mu and beta power decreased in the fronto-central region, but this decrease began earlier and was stronger when listening to vocal melodies compared to non-vocal melodies. Interestingly, the size of this “humanness” effect was inversely correlated with participants’ vocal accuracy.

### Beta and mu ERD during singing voice production

 While mu and beta ERD during the execution of limb movements have long been described, Gunji et al.’s study [31] was the first showing that a similar ERD occurs during singing or humming. Our present results confirm these data, suggesting that the relatively subtle movements of respiratory, laryngeal and labial muscles involved in singing on a vowel also generate this typical mu and beta ERD. The MEG source localization in Gunji et al.’s study [31] showed that ERD at 8-15Hz during humming resulted from the involvement of bilateral premotor areas, superior (trunk area) and inferior (face and larynx areas) part of the sensorimotor areas, secondary somatosensory areas, inferior parietal lobe-operculum and superior parietal cortex during singing and humming. ERD in the 15-30Hz frequency band resulted from involvement of a similar network with a larger implication of the sensorimotor cortex, from the superior to the inferior part. 

### Stronger motor activity during singing voice perception than during non-vocal melodies perception

The major result of the present study is that, in the fronto-central region, a mu and beta ERD occurred earlier during the perception of vocal models compared to the perception of non-vocal models. In the centro-parietal and parieto-occipital regions on the contrary, we observed an ERS in the mu frequency band in both listening conditions, possibly due to task induced modulations in arousal and increase in power of the alpha rhythm. The earlier mu and beta ERD for vocal models is likely to be linked to an anticipated motor preparation. It is indeed known that any voluntary movement is preceded by a motor activity indicating motor preparation, also measurable in the mu and beta bands. An anticipatory mu and/or beta ERD has been described several times (e.g [48, 26, 27]) 1 to 2 seconds prior to the movement or the go-signal. In the present study, the beta and mu ERD observed during non-vocal melody listening began 2 seconds before the go-signal, and thus corresponds to a typical motor preparation to singing. By contrast, mu and beta ERD during vocal melody listening began much earlier (4 seconds before the go-signal) than in the non-vocal condition, which means that hearing a vocal model could pre-activate the motor representations necessary to produce the sound well before the typical action preparation. This pre-activation may comprise automatic motor representations, voluntary preparation and attention-mediated processes which are also known to influence beta and mu rhythm [49-51].

Importantly, this earlier ERD to human voice perception cannot be attributed to a different working memory load, insofar as auditory working memory was equally required during non-vocal and vocal melodies listening. This effect does not seem to be linked either to a greater level of difficulty for vocal melody encoding and repetition, possibly engendering a greater preparatory effort. Indeed, our behavioral data do not suggest that the vocal condition was more difficult. As a matter of fact, several studies have demonstrated that vocal models are easier to reproduce than non-vocal ones [52, 53], especially for poor singers [54]. Although we did not find any significant behavioral advantage for voice stimuli in the present study, this is possibly due to the fact that poor singers were not represented enough in our sample (see the individual vocal accuracy measures Figure 5). This early motor activity during vocal model perception is thus more probably linked to the “humanness bias” described for action observation in EEG, fMRI or behavioral studies [55-57]. These works demonstrated that, compared to a non-biological model, perceiving a biological model induces stronger motor representations and facilitates imitation. In the auditory domain, Galati et al.’s fMRI study [18] has already suggested that different human-produced sounds, such as voice, may preferentially activate an auditory-motor network, compared to environmental sounds. This fMRI study concomitantly showed shorter reaction times for classifying human-produced sounds compared to non-human sounds.

This tight link between voice and sensorimotor representations is also supported by the source localization of the humanness effect in mu and beta frequency bands (Figure 3). Indeed, the region that seems to best explain the EEG rhythm differences during perception of vocal and non-vocal stimuli is a region in the somatosensory cortex. The localized source is left-sided, in line with the left-sided humanness effect presented in the results section for beta, and is slightly more dorsal than the larynx motor area localized by Brown et al. [58]. A further MEG study using MRI anatomical images for each subject will be needed to precisely localize the most sensitive region to the humanness effect. 

Finally, we found a significant correlation between the effect of humanness in the beta band and participants’ vocal accuracy. This effect suggests that the more singing difficulties participants had, the more their motor system was pre-activated when listening to the vocal models. This might be surprising in light of previous fMRI studies showing that motor resonance during perception is enhanced in experts (e.g. [59, 60]). Nonetheless, the result we report is more about impaired skills than expertise, and has to be interpreted in a perception-for-action context. One interpretation is that poor singers relied more on motor representations of vocal sounds to process the sound model and prepare their imitative gesture. This interpretation would be consistent with the dual-stream model developed by Hickok and collaborators for speech perception [61-63]. These authors have proposed that the dorsal auditory-motor circuit is required during vocal development and, in adults, in contexts where perceptual processing is getting more difficult because of noise or working memory load, while this pathway is not used for speech processing in normal speech listening conditions. We propose that people with a good vocal control are able to process the target sound and prepare the imitative vocal gesture thanks to auditory representations of the sound model, whereas people who are not used to doing such a task rely more strongly on kinematic properties extracted from the sound model. This latter group would thus be more sensitive to a human stimulus, containing biomechanic cues, compared to a non-vocal stimulus. 

In conclusion, this study has provided insight into the evolution of beta and mu rhythms during a vocal imitation task. Our data show that a singing task induces a beta and mu ERD, beginning during the perception of the sound models in the fronto-central region, earlier during the vocal melodies than during the non-vocal melodies. This result is in line with the bias for human stimuli described in paradigms of action observation/execution. Functional significance of this early motor activity during voice perception remains to be better defined in further investigations.
